# Chronotope Disruption as a Sensitizing Concept for Understanding Chronic Illness Narratives

**DOI:** 10.1037/hea0000151

**Published:** 2014-09-08

**Authors:** Tim Gomersall, Anna Madill

**Affiliations:** 1 School of Health & Related Research, University of Sheffield; 2 Institute of Psychological Sciences, University of Leeds

**Keywords:** diabetes, self-management, illness narratives, qualitative methods, chronotope

## Abstract

***Objectives:*** This article aims to elaborate chronotope disruption —a changed relation to time and space— as a sensitizing concept for understanding chronic illness narratives. ***Methods:*** Sixteen men and 16 women with Type 2 diabetes were purposefully sampled. Each was interviewed about his or her experience of diabetes self-management using the biographical-narrative interview method. Transcripts were inspected for key moments defined as emotionally laden stories relevant to the purpose of the research. We present dialogically inflected discursive analysis of exemplar extracts. ***Results:*** The analysis demonstrates how the concept of chronotope disruption helps identify, and understand, important aspects of patients’ chronic illness narratives. First, we investigate how medical advice can conflict with embodied experience and how progressive bodily deterioration can provoke a reevaluation of past illness (self-mis)management. Second, the increasing temporal and spatial intrusion of chronic illness into participants’ lives is examined. Finally, we focus on the masquerade of health as an attempt to manage, hide, or deny that one is physically challenged. ***Conclusions:*** Chronotope disruption offers a useful sensitizing concept for approaching chronic illness narratives and around which to organize analytical insights and to develop practice. Chronotope analysis fills an important gap in the science through compensating current health sciences’ focus on rationality, cognition, and *prospective* time (prediction) with a patient-oriented focus on emotionality, embodiment, and *retrospective* time (nostalgia). Chronotope disruption could be used to develop practice by gaining empathic understanding of patients’ life-worlds and provides a tool to examine how new technologies change the way in which the chronically ill have “being” in the world.

In this article, we elaborate a little-investigated aspect of chronic illness narratives: the grounding of biographical disturbance in time and space. Following the work of Bakhtin (1981, 1984), we identify this phenomenon as “chronotope disruption.” Chronotope refers to the way in which recognizable patterns of interwoven time and space are expressed in literature through genre forms (e.g., the way in which classic epic is set in the deep past, in dramatic locations, and stresses hierarchical social structure). According to Bakhtin, genres portray particular ideologies, in large part, through their representation of time and space (e.g., the heroic vision of epic). Thus, chronotope refers not merely to the formal features of a given narrative; of greater interest is the way in which chronotope defines a field of possibilities available to the narrator (Morson, 1994). Ideology includes assumptions about what counts as knowledge and our exploration of chronotope disruption will be illuminated by Bakhtin’s (1993) articulation of two key contrasting epistemologies: *istina* (abstract knowledge) and *pravda* (lived truth that is embodied and invested in emotionally). Chronotope helps structure the stories we tell about our everyday lives (Bakhtin, 1981), including, as we will show, narratives of becoming and being chronically ill.

Bakhtinian concepts have only recently been taken up in qualitative methods in psychology (e.g., Sullivan, 2012) and applied in health-related fields (e.g., Gomersall, Madill, & Summers, 2012; Madill & Sullivan, 2010) and have massive untapped potential. We argue here that chronotope disruption offers a particularly useful sensitizing concept (Glaser, 1996), or theoretically informed starting point, for approaching chronic illness narratives and around which to organize analytical insights and to develop practice. The kind, or pattern, of chronotope disruption communicated by patients will depend, at least in part, on the nature of their conditions and the way in which these are experienced and interpreted. In this respect, chronotope analysis bears some resemblance to the self-regulation model of illness, which emphasizes the role of visceral experience and personal interpretations of illness in guiding health-related action (e.g., Baumeister & Vohs, 2007; Leventhal, Brissette, & Leventhal, 2003). However, chronotope analysis goes further by exploring the ways in which making sense of “being ill” unfolds in the changing relationship between body, time, and space. For example, in terms of time, curable acute illness may lend itself to narratives structured by *restorative* time (regaining health), disabilities incurred through accidents by *ruptured* time (then–now), and chronic illnesses that are relapse-remitting to *cyclical* time (better–worse–better–worse).

In our elaboration of chronotope disruption in chronic illness, we focus on Type 2 diabetes as a chronic illness that tends to have increasingly negative impacts on health as time passes unless it is carefully managed. Here, as we will see, the course of the disease can be expressed in narratives structured by *provisional time* and, ultimately, by the revelation of *fading time*: both forms characterized by the *closing-in of time*, in terms of both scope (where time should be focused) and duration (how much time is left). In this respect, we show that chronotope poses some interesting answers to psychological questions around the agency and mutability of the self (see also Morson, 1994, pp. 86–88).

Narrative analysis has a long and sometimes vexed relationship with scientific psychology, stretching back to Freud’s case studies and Wundt’s *Volkerpsychologie* (Murray, 1997). However, use of the narrative form as material for psychological understanding has enjoyed a resurgence of interest in recent decades, particularly since Sarbin’s (1986) influential work proposing narrative as an alternative root metaphor for psychology. The intensity of narrative research requires relatively small samples and has often made use of the case study, or case study series, and Riessman (2003) notes that scientific disciplines tend to have an ambivalent relationship to this methodology. Narrative case studies are sometimes dismissed as anecdotal evidence, yet case studies are drawn on in teaching to convey technical knowledge through detailed examples and are increasingly recognized as an essential aspect of practice within health-related professions in order to facilitate appreciation of the lived experience of health and illness (Bell, 1999; Hydén, 1997; Mishler, 1984; Verghese, 2001). As Greenhalgh (2012) argues, while “hard” scientific knowledge is indispensable for the practice of medicine, so too is the clinician’s capacity for empathic understanding: “those who cannot feel will not see” (p. 95). Furthermore, when examined in detail, the majority of clinical cases fit the probabilistic and abstract discourse of evidence-based medicine quite poorly. Similarly, in terms of understanding health behavior, formal models can only go so far. The psychologist’s imagination and empathic understanding should also be enriched by attending to patients’ particular, concrete, day-to-day reality—the basis of the illness narrative form.

Narratives are important for understanding how people convey the changing relationship between body, time, and space brought about through illness (Radley, 1999). Narrative creates what Frank (1997) calls a “potential consciousness of illness” that makes suffering legible and relevant to both story teller and audience and is a means to engage with, and potentially reformulate, difficult experiences. Perhaps more familiar to health psychologists, Carr (1986) makes much of the link between temporality and meaning-making. Drawing on Husserl, he asks how we could lead meaningful lives if we experienced time as a series of isolated events. By relating a narrative we link up time to make coherent meaning out of the flux of experience (Murray, 2000; Ricoeur, 1984), a moment taking on its wholeness in relation to what preceded it and anticipated implications for the future.

The need to create coherence may, itself, be indicative of the disruption arising with the onset of illness. Bury (1982) developed an influential account of chronic illness experiences as forms of “biographical disruption,” in which the onset of illness is understood as “[a] kind of experience where the structures of everyday life and the forms of knowledge which underpin them are disrupted” (p. 169). For Bury, there are three dimensions to biographic disruption: the disruption of taken-for-granted assumptions and behaviors, the disruption to explanatory systems that necessitate a reevaluation of the self, and the mobilization of resources in response to the disruption. The biographical disruption model has generated a wealth of debate, discussion, and empirical research; has weathered postmodern and disability-movement critiques; and has provided a powerful argument for a focus on subjective embodiment in the social scientific study of illness (for a review, see Williams, 2000). Although scholars with an interest in biographical disruption do mobilize temporality and space in their analyses, this has typically been implicit and consequently underdeveloped. For example, Bury (1982) describes the loss for women with early onset rheumatoid arthritis as a shift “from a perceived normal trajectory through relatively predictable chronological steps, to one fundamentally abnormal and inwardly damaging” (p. 171). Here, benchmarks define where a body should be at certain temporal points in a life story, but Bury’s emphasis is the disruption to *biography* rather than the grounding of biographical disturbance in time and space that we are elaborating here as *chronotope* disruption.

More specifically, little empirical research has focused on temporal and spatial features in experiences of diabetes. Exceptions are Mol (2000), who investigated, from a Foucaultian perspective, the impact of Type I and 2 diabetic management devices on the experience of time; Lawton, Peel, Parry, and Douglas (2008), who investigated change in accounts of Type 2 diabetes causation over time; and Balcou-Debussche and Debussche (2009), who explored the facilitating aspects of hospital space-time on Type 2 diabetes self-management. Building on these studies of diabetes, we argue that sensitivity to the chronotope disruptions of chronic illness provides a theoretically informed starting point around which to organize analytical insights and to develop practice.

In summary, our purpose is to elaborate chronotope disruption as a sensitizing concept for understanding chronic illness narratives. Our example analysis is of the illness narratives of people diagnosed with Type 2 diabetes.

## Method

### Ethics

Ethical approval was granted by the Leeds Central Research Ethics Committee for research conducted in the National Health Service. Informed signed consent was obtained from all participants to take part in the study, to audio-record interviews, and to use anonymized quotes in reports. Participant pseudonyms are used throughout.

### Participants

Participants were recruited from two diabetes centers in a large city in the North of England. Sixteen men and 16 women took part, purposefully sampled within gender for equal numbers of poor and good glycemic control, as indicated by consistency of previous three recorded medical markers (HbA1c ≤7 and ≥10, respectively). We explore and compare the narratives provided by participants fitting these “good” and “poor” control profiles elsewhere (Gomersall, Madill, & Summers, 2012). Mean age was 65 years (range 45–81 years), and participants had been diagnosed with Type 2 diabetes for a mean of 14 years (range 2–30 years).

### Data Generation

Participants were each interviewed by the first author at home using an adapted version of the biographical-narrative interview method (Wengraf, 2001, 2009). This involved asking an initial question for inducing narrative: “Can you please tell me the story of your life since your first encounter with Type 2 diabetes, all the events and experiences that were important for you, personally, up to now? Please take as much time as you want.” The interviewer listened carefully and took notes that formed the basis of the second part of the interview in which participants were ask to elaborate on events, following the sequence in which the events had been related by the participant. Interviews lasted for a mean of 68 min (range 36–134 min) and were transcribed verbatim using the following conventions: . . . = *extract started or ended mid turn*; [. . .] = *text omitted midturn*; (.) = *very short, untimed pause*; (2) = *pause timed in seconds*; ((laughs)) = *description of additional communication information*; [racing course] = *names omitted for anonymity*; word underlined = *particular stress on that word*; dash at the end of a word = *a cut-off and change of direction midturn*.

### Analytical Assumptions

Dialogical analysis is sometimes grouped with other discursive and sociolinguistic methods that have been utilized in academic psychology in recent decades, including critical discourse analysis (Fairclough, 2011) and discursive psychology (Edwards & Potter, 1992). Like these approaches, dialogical analysis is skeptical with regard to the existence of enduring, internal psychological structures that pre-exist language (e.g., attitudes, beliefs, intentions). Instead, language itself is seen as constituting the psyche, which is, hence, observable through the deployment of linguist resources. However, whereas discourse analysis adopts a “blank” view of subjectivity (i.e., the author or speaker theorized as a text: Parker, 1994) or implies a strategic user of language (Madill & Doherty, 1994), Bakhtin assumes a “needy” subject, sensually engaging with the world and with others in a search for meaning (Sullivan, 2012). Hence, in terms of epistemology, dialogical analysis privileges *pravda* (lived truth that is embodied and invested in emotionally) over *istina* (abstract knowledge) and so rejects social constructionism in its strong form (Shotter, 1993).

### Analytical Procedures

Transcripts were inspected for key moments defined as emotionally laden stories relevant to this original purpose which had a recognizable beginning and end. Hence, key moments can be viewed as distinct episodes within the overall narrative structure of the interview (see Madill & Sullivan, 2010). In total, 186 key moments were identified, ranging between a quarter page to just over two pages of A4 in length, with participants contributing between two and 12 key moments each. Key moments were initially analyzed in terms of the following categories that operationalize central features of Bakhtin’s (1981, 1984) theory of chronotope: genre(s), emotional register, time–space elaboration, context. Notes pertaining to these four categories were written up into a table for each participant. While a variety of chronotopes were identified in the data (e.g., cyclical time, medical and lay timespaces), *chronotope disruption* was identified as an important phenomenon in that all participants described, in relation to their diabetes, increasing challenges in their embodied relationship with, or being in, time and space. Detailed familiarity with relevant key moments allowed us to identify three main patterns of chronotope disruption. We named each pattern in way that captured its central meaning. In the following section we provide an analysis of each identified pattern of chronotope disruption through close discursive analysis of exemplar extracts paying particular attention to time-space elaboration and emotional register (see Sullivan, 2012).

## Analysis

The analysis is presented in three sections, each demonstrating how the concept of chronotope disruption helps identify, and find a way into understanding, important aspects of patients’ chronic illness narratives, our example being Type 2 diabetes: (a) medicalized and embodied disruptions, (b) tyranny of the (body) clock, and (c) performing normativity.

### Medicalized and Embodied Disruptions

The concept of chronotope disruption fundamentally suggests a shift in one’s embodied ways of being in the world. However, in the early phases of a chronic illness, people may be asymptomatic and have to trust medical experts’ assurances that management is necessary.

Extract 1

Brian . . . Hand on heart I have never ever experienced anything faintly associated with diabetes until the surgeon or the nurse said, “There’s glucose in your urine. We’ll do a blood test. You could well be a diabetic.” And I’d not had a great thirst. I had not been tired. I had not been bad tempered. I’d not lost weight or put weight on. I’d stayed fairly stable. Nothing would have told me . . .

Brian lists the kinds of disruptions he might have expected with diabetes but had not experienced in its early stages: “a great thirst (. . .) tired (. . .) bad tempered (. . .) lost weight or put weight on.” He construes such signs and symptoms as being the body’s way of speaking, of communicating problems, but that his own body had been mute: “Nothing would have *told* me.” In fact, it is medical personnel, the “surgeon or the nurse,” who informed him of his illness through the results of a test, “said, ‘There’s glucose in your urine.”” And paradoxically he invokes the bodily idiom “hand on heart” to underscore how he had no prior knowledge of his illness when the reason had been his body’s very silence on the matter. Here the imperative for (self-)management arises from the abstract and scientific standpoint of the health professional, and Brian is asked to look beyond his own embodied experience.

Adrian, too, is requested to reconsider his accumulated embodied experience in favor of medical knowledge, but in the opposite direction to that of Brian.

Extract 2

Adrian I had a disagreement with Dr. X.

Interviewer Oh right yeah.

Adrian He said, “We want you down to about five,” and I said, “No way.” I said, “No way.” I said, “I start to hypo at six.” I said, “There’s no way you’re going to get me down to five.” He said, “Well that’s what we would like.” I said, “What you’re doing is you’re using it as a benchmark. You can’t do that because it affects different people different ways.” I said, “There’s no way we’re going to get me down to five.” So he said, “Well what are you happy with?” I said, “Between eight and fifteen I feel fine. Blood taken was about twelve point seven this morning. I’m quite happy at that. No side effects. I’m not sweating. No shakes. I’m quite happy at that” . . .

Whereas Brian had experienced no signs or symptoms, Adrian experienced his body as more vulnerable than the medical viewpoint would suggest: “No way [. . .] I start to hypo at six.” He also makes a case that the body is more unique and idiosyncratic than allowed for in medical knowledge arguing that the doctor is “using it [normative blood glucose values] as a benchmark. You can’t do that because it affects different people different ways.” Of particular interest is the way in which Adrian evokes blood glucose level as a specific kind of illness chronotope that provides the coordinates of his wellbeing over time: “between eight and 15 I feel fine.” Hence Adrian’s “disagreement with Dr. X” is an attempt to avoid, what would be to him, the chronotope disruption of “going down to about five” and likelihood of entering the traumatic timespace of a “hypo.” The felt body, here, is disrupted in an obvious or “noisy” fashion—“sweating [. . .] shakes”—in stark contrast to the disembodied and abstract “benchmark” constructed for him in medical space. Consequently, the experienced disruption of the hypo takes precedence over longer-term blood glucose control as advised by the doctor.

In this extract, Adrian describes a discrete past event but communicates it in reported direct speech inviting the felt immediacy of the original interaction: “He said [. . .] I said [. . .] I said [. . .] I said [. . .] He said [. . .] I said [. . .] I said [. . .] So he said [. . .] I said.” This has the effect of presenting his perspective as a still current truth. In this way, Adrian’s account fuses past with present, the form of his narrative cohering with his attempt to maintain the chronotope stability of his embodied experience within the context of tensions with medical advice.

For others, avoiding chronotope disruption was, in contrast, premised on a nostalgic maintenance of their prediabetes orientation to time and space. In the absence of immediately felt threat to health, several participants reported rejecting (self-)management at the asymptomatic stage. Brian, for example (Extract 1), went on to explain how he “stupidly paid it no respect whatsoever” until, after a number of serious complications, he received the strongly worded warning from his doctor that “you haven’t got a long time to live if you keep going like this.” A similar ultimatum is implied in the following extract from Rachel. Here we demonstrate how sensitivity to the ways in which chronotope disruption is woven into patients’ narratives can provide insight into important shifts in subjectivity and stance toward illness (self-)management.

Extract 3

Rachel I’ve learnt I don’t have to say yes to everybody. Because I was “yes yes” I would agree with- you could tell me I was black and I would have said “Yeah I’m black.” I’d agree with them. And then it was like over the years and then finally after this time and I realised no you don’t have to. So the rebellion started in and then- no I don’t think rebellion is the word. I think I’ve found my voice. So you’re telling me, “Rachel you’ve got diabetes. Now you’ve got to learn to control this sugar. You need to cut down. You need to cut these things out.” And I think, “Who are you to tell me what to do?” [. . .] What you need is the experience and to learn how to control to see what I got away without experiencing exactly what it can do to my body. And now over these last few years I’m seeing what it can do to me and pray to God I haven’t left it too late.

At the beginning of this extract, Rachel articulates a move away from a previous acquiescent, passive position through an extreme case formulation suggesting that she would have accepted even such blatant falsehoods as being told she was “black.” It is only with the passing of time, “over the years and then finally after this time,” that she “finds her voice.” Thus, in a similar way to Adrian (Extract 2), the voice emanating from *medical* space—“Now you’ve got to learn to control this sugar. You need to cut down. You need to cut these things out”—is rejected as an unwarranted imposition into her *personal* space. However, this intonation of defiance—of questioning the right of healthcare professionals to “tell [her] what to do”—is also rendered problematic from her current perspective: “over these last few years I’m seeing what it can do to me and pray to God I haven’t left it too late.” This indexes a transformed subjectivity: contrite, frightened, and seeking redemption, which, in turn, shows how chronotopes can build up and overlay one another in the course of a narrative (see also Holquist, 1990, pp. 116–121).

The anguish of potentially being “too late” suggests revelation of being on the edge between provisional time (in which there is still hope that one can *make provision*) and fading time (in which there is only a limited viable future for the self). Indeed, for many participants, felt changes in the body were a prerequisite for engaging with (self-)management and the warnings of medical professionals, friends, and family acquired a retrospective, nostalgic prescience as diabetic complications emerged. And we can see in Rachel’s narrative a wider arc of teleological time in that the meaning of past events becomes evident only from her standpoint in the present and, as in all these disparate examples, the invitation is to disavow the past.

Notably, each of the above extracts (1–3) relates specifically to medical space, and it seems the contrast between embodiment and medical knowledge could often create disagreements between our participants and medical professionals. However, as we go on to explore below, being ill in different spaces could be associated with some quite different subjective implications.

### Tyranny of the (Body) Clock

As it worsens, chronic illness can make increasing temporal and spatial intrusions into life. In particular, our participants described how managing diabetes could diminish their capacity to improvise in time. They had to monitor and restrict what they were ingesting, and when, and this vigilance could severely dent their sense of acting freely or spontaneously. Jacob explained:

Extract 4

Jacob Um I suppose really er (2) the um (.) particularly er my son lives in Australia [. . .] I’ve been over there a few times y’know [. . .] and of course it’s hot over there ((laughs)) [. . .] so it it’d be very nice to be able to just er y’know drop into er a bar somewhere y’know a club or something like that (.) and get a couple of pints y’know.

Interviewer Mm mm.

Jacob And it’s so easy to just sit down and say, y’know I’ll have a sandwich. I’ll have a packet of crisps. Something like that y’know [. . .] um I’m thinking have I done that (.) take more insulin ((laughs)) have I y’know. So it’s not entirely (1) out o’ way to do that y’know but you can’t do it without thinking . . .

Jacob states that “it’d be very nice to be able to just er y’know drop into er a bar somewhere y’know a club or something like that (.) and get a couple of pints.” In doing so, he construes this desire to be unremarkable: “to *just*,” and to consist of casual actions: “*drop* into. . .a bar *somewhere*. . .get a *couple* of pints.” And he repeats this structure, emphasizing the normality of his wish and the ease with which it usually associated: “so easy to just sit down and say y’know I’ll have a sandwich. I’ll have a packet of crisps.” These ordinary pleasures are not completely denied him. They are not “not entirely (1) out o’ way.” But the spontaneity has gone and it has become effortful in that Jacob now has to prepare himself, “take more insulin,” and “can’t do it without thinking.” Similarly to the extracts in our previous section of analysis, there is interplay here between Jacob’s desire to enjoy ordinary activities and the requirements of diabetes self-management. However, unlike the previous extracts, he does not attribute the imperative for self-management as emanating from an external, clinical space. Rather, he has incorporated it as part of his own “thinking.”

Our participants described the intrusiveness of the illness increasing as the diabetes progressed, especially in terms of demands on their time. This phenomenon was described evocatively by Lauren. She had been diagnosed with Type 2 diabetes for 12 years and deteriorating glycemic control had led to an intensive regime comprising insulin and tablets.

Extract 5

Lauren . . . I’m constantly taking medication and I just felt so fed up and there came a stage where I just- I didn’t want to get out of bed. I didn’t want to take my medication. I mean at one point I used to keep my tablets in a little box you know in open view. Now they’re in the kitchen in a drawer that is shut because I don’t want to look at them . . .

Lauren’s description of herself as “*constantly* taking medication” indicates the felt temporal pervasiveness of treatment. In fact, her narrative is full of temporal signifiers: “there came a stage,” “at one point,” “I used to,” and “now.” These present the progression through which medicines have taken over Lauren’s existence, and the overwhelming unpleasantness of this is conveyed by her attempted stasis of not wanting “to get out of bed.” To avoid the potentially fatal consequences of acting on the feeling that she “didn’t want to take (her) medication,” Lauren has had to distance herself from them. She used to keep her tablets “in open view” but now puts them “in the kitchen in a drawer that is shut because I don’t want to look at them.” Hence, despite the relatively small amount of literal space taken up by tablets that can be kept in “a little box,” Lauren feels that they are in danger of taking over her subjective world with the lines between personal and medical space blurring.

Living with chronic illness, then, can diminish the capacity to improvise in time, can pervade the experience of “being in time,” and dominate one’s personal space, leading some patients to develop strategies for reducing or coping with these chronotope disruptions. This may be especially pronounced in an illness such as diabetes that requires regular, vigilant monitoring and often profound changes in hitherto taken-for-granted practices. Participants, such as Jacob, offset preparation against the normalization and pleasure of eating “treat” foods or indulging at special occasions and others, such as Lauren, used creative approaches such as hiding medications from view. Others took a more fatalistic stance, adapting as best they could when the need arose and, perhaps paradoxically, in so doing, rescuing some sense of spontaneity. These orientations to the challenges of worsening chronic illness are not mutually exclusive and Thomas’s story incorporated a complex mix of adaptation, resistance, and fatalism: provisional time and fading time.

Extract 6

Interviewer Yeah (9) now you say um you say you’ve arrived at your present state that you’re happily checking your blood sugar every couple of days and er injecting your insulin and so forth. Um can you tell me about this phase? The present state?

Thomas Um I’m OK. It hasn’t stopped me doing anything I want to do.

Interviewer Mm.

Thomas I can still go to cricket matches. I can still go drive my motor car around. Go to [racing course] to the hill climb. Really the um the urgency has gone out of life because if I don’t do something today I can do it tomorrow which is nice. I enjoy that. Um (.) there is of course the inevitable um decline towards desperation and death um which I try not to think about. It’s gonna happen sooner or later (1) but then it’s going to happen to everybody isn’t it?

Interviewer Of course yeah.

Thomas Not just me fortunately. So you do um (1) worry about the future at times. It it’s completely out of control as far as I’m concerned. I can’t see that I can do anything about it . . .

This extract commences with the interviewer glossing Thomas’s narrative so far as indicating positive adaptation to a relatively intensive medical regime: “you’re happily checking your blood sugar every couple of days and er injecting your insulin.” The disruptive potential of this regime, but also Thomas’s success resisting this disruption, is indicated in his response that “(i)t hasn’t stopped me doing anything I want to do.” The importance of adaptation as a strategy of resistance to the encroachment of illness is stressed by Thomas through repetition in a three-part list of what it is that he *can* do: “go to cricket matches [. . .] go drive my motor car [. . .] go to [racing course]”. And that he can “still” do these things indicates a nostalgic desire to preserve these pleasures against the threat that they will be lost as time passes. However, there are limits to his personal agency. Luckily, as a retired person, “if I don’t do something today I can do it tomorrow.” But this suggests accepting and working round disrupted plans, or making always provisional plans, due to his illness. In more radically deterministic terms, the temporal association in the narrative to an unavoidable finality—“there is of course the inevitable um decline toward desperation and death”—suggests a *foreshadowed* time (Morson, 1994) in which the possibilities of the present are held hostage, limited in their meaning by a predestined future.

Hence, in Thomas’s extract there is an exploration of where he can, and cannot, muster control over the impact of his progressing disease—or to put it the language of chronotope, to what extent he can view timespace as open or closed. Importantly, this is infused with contrasting affective valance. Thomas does not challenge the interviewer’s description of him as “*happily* checking your blood sugar.” In his own words he is “OK” and he implies a pleasant serenity in his current circumstances in which he has agency, even if it is constricted to choosing *not* to act: “if I don’t do something today I can do it tomorrow which is nice.” On the other hand, the future, construed as out of his control, is saturated with distressing emotions, which suggests continued resistance within his apparent fatalism: “*inevitable* um decline toward *desperation* and death um which I *try not to think about*” and “*worry* about the future at times. It it’s *completely out of control.*” Thomas is making the best of provisional time, grasping a sense of agency and maintaining pleasant activities where he can. However, time is closing in, the (body) clock is still ticking, and its tyranny infuses Thomas’s account.

### Performing Normativity

It is recognized by scholars (e.g., Swain & French, 2008) that many of the problems encountered by disabled people arise not strictly from the body but from an environment that assumes able-bodiedness—and that thus constitutes a space often difficult to negotiate when one does not conform. One participant, Caroline, gave an evocative example of such a disruption. Along with Type 2 diabetes, she had experienced a lupus infection in her lungs, which additionally compromised her mobility. In the following extract, she describes an inventive strategy to perform normativity in spite of the limitations of her body.

Extract 7

Caroline . . . but gradually I find when I finished working at the end of the day- I used to find at the end of the day where I’d parked the car in the car park. It took me ages to get there because I was so breathless. So what I used to do is buy an evening paper. Pretend I’m reading it and puff a little bit till I get to the car. And then it got worse and worse and I thought, “Something’s not right here.”

Caroline’s account could be viewed as a chronotope metamorphosis because the changes she noticed were incremental, appearing “gradually” and getting “worse and worse,” and eventually having a significant impact on mundane activities such as walking to her car: “took me ages to get there because I was so breathless.” She then goes on to describe how she managed this disruption: she used to “buy an evening paper pretend I’m reading it and puff a little bit till I get to the car.” Interestingly, what she attempts to mitigate is the potential negative evaluation of her breathlessness by strangers in the car park—thus enacting a protection of “face” (Goffman, 1955) via a manipulation of time and space. Although she does not fulfil the ideal of the healthful body, she can *perform* this body—masquerade as healthy—in the act of “pretend(ing)” that her slow movement is due to reading the paper. However, maintaining this façade of normality is increasingly effortful and, as her puffing gets “worse and worse,” she comes to the realization that “something’s not right here.” This raises the possibility that manipulating her social presentation, that is, attempt to hide the chronotope disruption of being physically challenged through chronic illness in the presence of others, is pending also her own engagement with her physical problems.

As Caroline’s anecdote suggests, adjusting to degeneration in bodily functionality was often most difficult in terms of one’s ways of being with others. This also became complex for some of our male participants who struggled to find new ways of doing gender in the context of a chronic illness that limited their capacity to inhabit traditionally masculine timespace. Brian, for example, spoke about a profound affective change that had set in following a heart attack.

Extract 8

Brian I can watch the television and summat and I start crying. There’s tears rolling down me face. That’s happened for last ten years but it’s gradually got worse y’know. Somebody that knows me they says, “He couldn’t have a heart attack ’cause he didn’t have a heart.” And I were known as destroyer when I played rugby y’know. Nobody were too big. Nobody were too tall and I never (.) all I used to think about were winning and I I never had no no qualms wi’ what I had to do. I I were a destroyer and they had a destroyer. Every team had a destroyer. Everybody had an enforcer [. . .]

Interviewer Mm mm (5) that sounds like quite a big change in your in your experience there.

Brian Well it is ’cause it can happen when you’re out as well [. . .] y’know. And if it just happened when I’m at home I wouldn’t take no mind but it can happen when I’m out.

Like Caroline’s walk to her car, Brian describes bodily changes that “gradually got worse” in relation to a mundane, everyday space, this time watching “the TV.” However, unlike Caroline’s breathlessness, the change that Brian describes is emotional, gendered, and counterhegemonic: he is a man who can “start crying” while watching TV. This appears to be a longstanding change in that is has “happened for last 10 years” and is one that is presented as a complete contrast to a former hypermasculine identity.

He has been a rugby player “known as destroyer” due to his physical prowess, and his masculinity was enacted through being competitive and ruthless: “all I used to think about were winning and I I never had no no qualms wi’ what I had to do.” Thus, chronotope is disrupted in that Brian can no longer move through the time and space of the rugby pitch in ways that enact a “destroyer” identity. On the contrary, he encapsulates the shock associated with his new affective vulnerability in the evocative statement that “he couldn’t have a heart attack ‘cause he didn’t have a heart,” although credits this poetics to “somebody that knows me.” However, he de-emotionalizes his crying so that, although “there’s tears rolling down me face,” no affective trigger is specified. The tears just “start”; it can just “happen” when he is out, and “just happened when I’m at home.” Importantly, though, whereas in private his tears are of little consequence: “I wouldn’t take no mind,” making the contrast with crying that “can happen when I’m out,” implies that this change does bother him in social space.

Men were more likely to lament how chronic illness had forced them out of work, or to adapt through taking up different work, underlining how chronotope generally, and chronotope disruption specifically, can be a gendered phenomenon. Adam, for example, explained that he had to give up the manual side of plumbing to focus on the administrative side of his business. He could still clear drains because “there’s no finesse in it,” but plumbing that required “dexterity” was no longer available to him due to loss of feeling in his fingers. Hence, like Caroline, a particularly difficult but subjectively important aspect of chronic illness for some people is presenting as operating still in the timespace of healthy others—particularly for men, this being a hegemonically gendered space—as a way of managing, hiding, or denying the chronotope disruption of chronic illness.

## Discussion

Our purpose in this article was to elaborate chronotope disruption as a sensitizing concept for understanding chronic illness narratives, the example being people diagnosed with Type 2 diabetes. Our analysis demonstrated three ways in which chronotope disruption helped identify, and find a way into understanding, important aspects of patients’ chronic illness narratives. First, in Medicalized and Embodied Disruptions, we investigated chronotope disruption in terms of how medical advice can conflict with embodied experience and how progressive bodily deterioration can provoke a reevaluation of past illness (self-mis)management. Second, in Tyranny of the (Body) Clock, we explored chronotope disruption in terms of the increasing temporal and spatial intrusion of chronic illness into the participants’ lives. Finally, in Performing Normativity, we focused on the masquerade of health as an attempt to manage, hide, or deny the chronotope disruption of being physically challenged through chronic illness, particularly in the presence of others. All are infused by a retrospective nostalgia for a being-in-the-world that has forever gone. We now discuss the potential of chronotope disruption to fill an important gap in the science and to impact the way we approach chronic illness: first as a concept around which to organize analytical insights, considered with respect to each of the three strands of our analysis, and second, more broadly, as a concept around which to develop practice.

### Medicalized and Embodied Disruptions

Although chronic illness typically cannot be cured, it can be managed. This places responsibility on the patient to implement medical advice, and facilitating adherence is an important aspect of health psychology research and practice. We have shown how sensitivity to chronotope disruption reveals the often clashing timespace, and hence “truths,” of patient and practitioner, provides insight in why some patients may not engage with (self)-management. Bakhtin has identified these distinct orientations to truth as *pravda* (subjective experience) and *istina* (objective abstracts): “(*p)ravda* is not only context specific and unique, but also embedded in the actual act of a specific person. *Istina,* to the contrary, is composed of universal moments” (Sidorkin, 2004, pp. 6, italic in original). From the medical point of view, illness (self-)management practices are based on objective evidence, and engaging with them is rational and inherently desirable. In contrast, patients oriented to their individual, situated embodiment, and emotional investments that were not always conducive to following medical advice.

The contrasting epistemologies of *istina* and *pravda*, which is an important aspect of Bakhtin’s theory of chronotope-as-ideology, helps reveal tensions in the notion of “control” as a central feature of chronic illness management that further illuminates patient resistance. Using diabetes, Broom and Whittaker (2004) show that “control” has a double meaning. First, control can be used in a more or less straightforward biochemical sense to denote the level (position in space) and stability (over time) of glucose in the blood (*istina*). Second, it can refer to a patient’s ability to regulate their own actions: their “self-control” (*pravda*). In the case of “lifestyle” illnesses, such as Type 2 diabetes, these two notions of control can become conflated in an implicit—or feared—moral (self-)assessment of the patient’s worth (that is, ability to self-control and, hence, to be a good patient and citizen) through the measurement of glucose levels. Furthermore, the control denoted by tests (*istina*) can usurp the patient’s sense of self-knowing (*pravda*) when, as Karas Montez and Karner (2005) suggest, “some physicians teach their patients that with respect to how they feel, the numbers or diagnostic images are more reliable, resulting in a distrust of subjective feelings” (p. 1095). Indeed, as we identified in relation to Adrian (Extract 2), Mol (2000) elaborates how test results from self-monitoring can become for some patients a specific illness chronotope demarcating their relative well-being or fragility.

The contrasting epistemologies of *istina* and *pravda* are also relevant to understanding the concept of chronotope disruption as an important counterbalance to the current, dominant cognitive paradigm within health psychology (e.g., the theory of planned behavior: Ajzen, 2011), which has been critiqued as overly rational (e.g., Dutta-Bergman, 2005). In fact, when psychologists attempt to measure personality or health cognitions, and tie such constructs in with health outcomes via the language of probability, they can be understood as engaging in ontological procedure similar to the authors of the classical adventure. Here, the hero’s preexisting and unchanging character (stoical, wise, fearless) determines the outcome in advance, while time is reduced to an empty series of relatively independent happenings that the hero merely happens to pass through (Bakhtin, 1981; see also Holquist, 1990, pp. 109–110). Such an assumed transcendence of the mind has a long history with roots stretching at least as far back as early Western metaphysics (Hope, 2011).

In contrast, emotional register is an inherent feature of Bakhtin’s theory of chronotope, which forefronts also the pleasures and pains of the flesh as lived. Moreover, sensitization to the concept of chronotope allows to see the prioritization of the future and, hence, of *prospective* time in medical and health sciences concerned with prediction and control. And, as our analysis shows, this can be at odds with patients’ search for *meaning*, which, poignantly, tended to emerge *retrospectively* in an anguished nostalgia and only once the link had been experienced viscerally between past (in)action, the deteriorating body, and the imminent *end* of the(ir) future. Hence, there is a strong argument that chronotope as a sensitizing concept is important in illuminating a blind-spot in current health psychology identified reflexively through analyzing the timespace mismatch that can exist between the current, dominant research paradigm and the patients that health psychologists are seeking to understand.

### Tyranny of the (Body) Clock

All our participants talked about the increasing temporal and spatial intrusion of chronic illness into their lives. In particular, we considered this in relation to the compromise of improvisational temporality. The provisionalization of time may be greatest where the most extensive illness management efforts are required and the structured temporality of self-management was ubiquitous in our participants’ narratives. However, paradoxically, the medications and technologies of self-monitoring that help structure time also enable patients to improvise in time and a number of participants spoke about learning to use insulin responsively to their needs, of transgressing occasionally, or, quite simply, their unproblematic relationship to the demands of self-management.

Similarily, Balcou-Debussche and Debussche (2009) demonstrate how bodily transition into a context of provisional time (i.e., hospitalization) could be beneficial for self-management practices. Balcou-Debussche and Debussche conceptualize hospitalization for people with diabetes as a “suspension of reality,” in which time and space become highly structured with medical professionals on hand and diabetes-friendly meals provided. As a rupture in mundane timespace, hospitalization can be considered an exemplary instance of provisionalized time. However, aspects of this experience assisted illness control and consequently were valued by Balcou-Deussche and Debussche’s participants. Hence, we must avoid assuming that timespace disruption associated with chronic illness is always burdensome. Indeed, as our analysis demonstrates, people are often very inventive when it comes to adapting to change after the onset of illness. The experience of temporal improvisation may even be heightened for people with chronic illness insofar as it provides a counterweight to the objectification of time often involved in self-care (Morris, 2008).

The concept of chronotope does, however, allow us to understand some patient behaviors as creative management of situated timespace disruption rather than, more negatively, disengagement with healthcare advice. Moreover, we argue that it has advantages over extant organizing concepts characterizing illness narratives. For example, Bury’s (1982) concept of *biographical disruption* has been critiqued for overly stressing discontinuity (Williams, 2000), with Pound, Gompertz, and Ebrahim (1998) finding that the onset of stroke in older working-class people in the East End of London was perceived to be a *continuation* of their life struggles. Chronotope disruption captures at a higher level than biography the organization of meaning, and does not assume that fundaments of a life narrative will change with the onset of chronic illness, while being sensitive to the inherent chronicity (time) and embodiment (space) of long-term illness, however it is storied.

### Performing Normativity

Our analysis focused on the masquerade of health as a nostalgic attempt to manage, hide, or deny the chronotope disruption of being physically challenged through chronic illness. Disruptions could be managed, for example, through allowing oneself “treats” during special social occasions. However, as physical impairments encroached on activities, they evinced a greater tendency to mask or deny limits through performing a “normal” body. Puar (2009), drawing on Butler (1990), suggests how the concept of performativity may be extended to consider how disable people live in a society of compulsory able-bodiedness. Furthermore, masquerades of health can be understood as a protection of “face” (Goffman, 1955), in which one attempts to accomplish a desired social identity. And our participants’ attempt to avoid social devaluation was amplified for men who eschewed the way in which chronic illness could undermine characteristics associated with hegemonic masculinity (e.g., toughness, invulnerability, independence; Courtenay, 2000).

Chronotope disruption associated with chronic illness can, therefore, mean being “out of step” and “out of time” with the social life-world of healthy others. It can also mean being “out of joint” with oneself, even if “passing” (Butler, 1990) is achieved to the extent that, like Caroline (Extract 7), one is so immersed in the “spatiality of situation” (Merleau-Ponty, 1962/2002) that the act feels real—that is, until the performance becomes too effortful: “And then it got worse and worse and I thought, ‘Something’s not right here.’” Hence, becoming chronically ill can mean negotiating a newly queer identity, betwixed and between, slipping between categories, and with the potential of being devalued into self-denial. The concept of chronotope disruption therefore has potential also to foster understanding and critique of the lived environment and the social landscape from a novel concern with *affective timespace*, which includes the impact and internalization of the gaze (Foucault, 1977). In terms of theory, Bakhtin preempted many aspect of poststructuralism and, hence, allows fruitful connections with contemporary gender and queer theory—as illustrated here—and has potential for informing also psychoanalytic approaches psychosocially conceived.

### Implications for Practice

We have examined how the chronotope of people with chronic illness, our example being Type 2 diabetes, can differ, often profoundly, from that of the able-bodied, the health practitioner, and the current, dominate research paradigm in health psychology. An important implication for practice is to consider how to approach and support people with chronic illness from an understanding of, and respect for, *their* perspective and this is a central ethos of patient-centered care (Kitson, Marshall, Bassett, & Zeitz, 2013). As the medical profession is coming to realize (e.g., Greenhalgh, 2012), patient narratives are essential for understanding particularity of need and everyday health-related practices and provide a complementary type of knowledge to those aspects of practice that emphasize measurement, standardization, and aggregation. More specifically, insofar as narratives *always* signpost some kind of structure of time and space (Carr, 1986; Murray, 2000), sensitivity to chronotope disruption can help practitioners couch self-management advice and offer strategies that takes into account patients’ own temporal and spatial world, acknowledging creative compromise, and finding common ground that allow both to move toward each other.

Chronotopes associated with chronic illness, as in all contexts, are historically embedded. This is not only due to the rapidly changing and expanding array of usable narrative forms that have proliferated in our postmodern epoch (Gergen, 1991), but also to the rapid development of medical technologies that have implications for health practice. For example, a new generation of pumps has become available for people with Type 1diabetes that administer fast-acting insulin and offer relative freedom from the structured diet and exercise regimes usually involved in self-management. There is even a possibility that, if combined with a continuous blood glucose monitoring system, an insulin pump could function as an artificial pancreas (Jacobs et al., 2011). Such technologies could have profound implications for temporal-spatial experience in diabetes self-management and early trials already indicate greater satisfaction and quality of life (McMahon et al., 2005). However, as Mol’s (2000) analysis of blood glucose monitoring shows, technology, while potentially providing a more comfortable life, can bring unintended consequences, for example in terms of increased surveillance and control (Foucault, 1977). Exploring these and similar new healthcare technologies is likely to be an important area of research for health psychologists, and qualitative methods elucidating the experience of time and space could help recognize and suggest strategies to minimize their negative, subjective impact.

## Conclusion

There is no singular and straightforward way in which a given illness is narrated and self-understood in terms of chronotope. This depends on contingencies including life context, former relationship with timespace, and the medical and psychosocial resources available for dealing with disruptions. Qualitative methods are ideally placed to capture such complexities and to explore how apparently similar physiological manifestations of illness can be storied in different ways (Madill & Gough, 2008). Nevertheless, our analysis did also identify some commonalities between participants. Living with chronic illness can be predominantly emotional and visceral (as opposed to approaches in health sciences that tend to stress cognition and rationality), can diminish the capacity to improvise in time as well as pervade the experience of “being in time” and can change one’s sense of self in further unsettlingly “queer” ways through being subject to, and internalization of, the evaluative gaze. These may be especially pronounced in illnesses such as diabetes that require regular, vigilant monitoring, and often profound changes in social practices. A program of research could identify characteristic chronotope disruption associated with different kinds of illness and provide insights into new ways of promoting healthy practices and providing patient-centered care. Heterogeneous types of data, such as field-notes or patient’s photographs and drawings, could be analyzed for chronotopic insight, and the focus on our material existence in timespace suggests innovative trans-disciplinary cross-fertilization with research in geography, architecture, and environmental sciences.
